# Site-Specific Sequence Exchange Between Homologous and Non-homologous Chromosomes

**DOI:** 10.3389/fpls.2022.828960

**Published:** 2022-02-03

**Authors:** Qian Yin, Ruyu Li, David W. Ow

**Affiliations:** ^1^Plant Gene Engineering Center, Chinese Academy of Sciences Key Laboratory of South China Agricultural Plant Molecular Analysis and Genetic Improvement, South China Botanical Garden, Chinese Academy of Sciences, Guangdong Key Laboratory of Applied Botany, Guangzhou, China; ^2^University of Chinese Academy of Sciences, Beijing, China

**Keywords:** gene stacking, transgene replacement, transgene translocation, GMO, recombinase, Cre

## Abstract

Transgene integration typically takes place in an easy-to-transform laboratory variety before the transformation event is introgressed through backcrosses to elite cultivars. As new traits are added to existing transgenic lines, site-specific integration can stack new transgenes into a previously created transgenic locus. *In planta* site-specific integration minimizes the number of segregating loci to assemble into a breeding line, but cannot break genetic linkage between the transgenic locus and nearby undesirable traits. In this study, we describe an additional feature of an *in planta* gene-stacking scheme, in which the Cre (control of recombination) recombinase not only deletes transgenic DNA no longer needed after transformation but also mediates recombination between homologous or non-homologous chromosomes. Although the target site must first be introgressed through conventional breeding, subsequent transgenes inserted into the same locus would be able to use Cre-mediated translocation to expedite a linkage drag-free introgression to field cultivars.

## Introduction

Crop development *via* transgenesis is typically done by inserting DNA into an easily transformable variety and then introgressing the transgene out to many different locale-specific cultivars. As new traits are developed, it becomes a challenge of where to integrate new trait genes. If inserted into a new locus, breeders will have more loci to reassemble back into a breeding line. Efforts to cluster multiple transgenes at a single integration locus can be achieved through prior stacking of the many genes *in vitro* into a single plant transformation construct (Goderis et al., 2002; Chen et al., 2006; Shih et al., 2016; Zhu et al., 2017; Collier et al., 2018). However, relying solely on this approach means that further addition of transgenes would require combining new genes with previously introduced transgenes into a larger transformation vector. Though this is not a technical limitation, it could have legal ramifications. Previously introduced traits could then require a new round of de-regulation for being a new transformation event. Adding more transgenes to an existing transgene locus is possible *via in planta* site-specific integration through the use of site-specific nucleases (Puchta et al., 1993; Wright et al., 2005; Fauser et al., 2012; Zhang et al., 2012; Dong and Ronald, 2021) or site-specific recombinases (Albert et al., 1995; Day et al., 2000; Li et al., 2009; Hou et al., 2014; Nandy et al., 2015; Chen and Ow, 2017; Chen et al., 2019). This would insure co-introgression to field cultivars without additional loci to impede downstream breeding.

**FIGURE 1:**
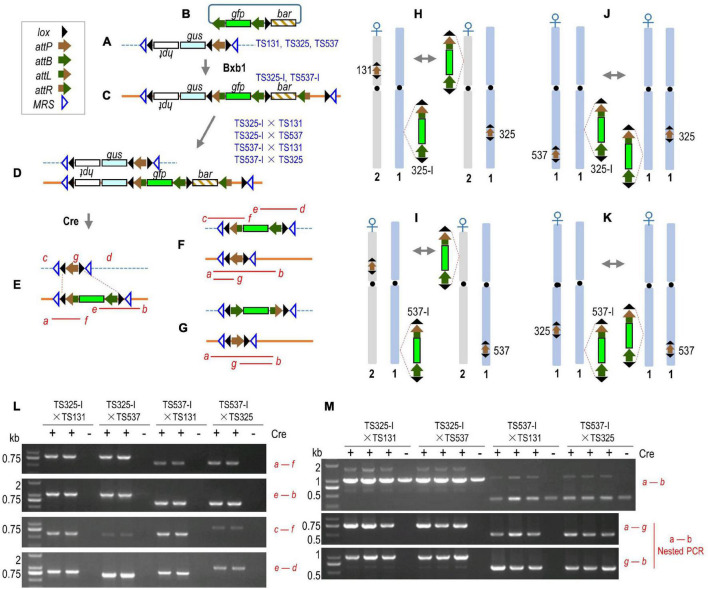
Cre-mediated recombination between different target sites. **(A)** Rice target lines TS131, TS325, and TS537 structure. **(B)** Integrating vector pZH210B was inserted into TS325 and TS537 to form TS325-I and TS537-I, respectively **(C)**. F1 hybrids from crosses **(D)** and expected structures from Cre-mediated deletion of DNA flanked by directly oriented *lox* sites **(E)**; inversion from recombination of oppositely oriented *lox* sites not shown. Translocation of *gfp*-containing segment to another chromosome **(F)** and after inversion **(G)**. Red italic letters, PCR primers; red lines, PCR products. **(H–K)** Chromosome location of target sites in TS131, TS325, and TS537 with relevant *lox* sites and the direction of *attP*. Crosses with *gfp*-stacked lines (i.e., 325-I and 537-I) leads to Cre-mediated reciprocal translocation of the *lox*-flanked DNA. **(L,M)** PCR results from deletion and chromosome recombination in F1 + *cre* plants. Genes described in the Methods section and were transcribed in direction of lettering, promoters, and terminators not shown. Recombination sites indicated in inset legend. DNA size is mentioned in kb.

We had described an *in planta* gene-stacking system that uses mycobacteriophage Bxb1 integrase (recombinase) for site-specific integration (Hou et al., 2014). In this system, a target site is first created by the insertion of a first trait gene linked to an *attP* (phage attachment) sequence that serves as a “target site” (Figure 1A). New DNA is introduced through a donor construct, such as a second trait gene plasmid that also carries two complementary *attB* (bacterial attachment) sequences (Figure 1B). The recombination of one plasmid-encoded *attB* with the genomic *attP* places the incoming DNA precisely into the genomic target (Figure 1C). Since the donor DNA can carry two *attB* sites, two configurations are possible depending on which *attB* site recombines. Figure 1C shows the preferred configuration that can be screened by polymerase chain reaction (PCR), and the *attB* not used in the first round of integration can serve as a target site for the next round of integration by a donor plasmid with two *attP* sites (not shown; refer to Hou et al., 2014). In theory, this permits serial gene stacking by alternating between the uses of *attB* and *attP* donor plasmids. Cre (control of recombination) recombinase is then introduced to delete away transgenic DNA flanked by *lox* (locus of *x*-over) sites that is no longer needed after transformation (Figure 1E).

Of particular relevance is that the target construct has been designed with a set of *lox* sites in the opposing orientations (Figure 1A), in which they are retained after Cre-mediated deletion regardless of the number of transgenes stacked into the target site (Figure 1E). This pair of inverted *lox* sites can serve as recombination substrates for Cre-mediated intra-chromosomal inversion, as well as inter-chromosomal recombination. Prior studies have shown that Cre is capable of causing recombination between different chromosomes (Qin et al., 1994; Smith et al., 1995; Koshinsky et al., 2000; Vergunst et al., 2000; Zong et al., 2005; Titen et al., 2020). Inter-chromosomal recombination would break genetic linkage that could potentially expedite transgene introgression from a laboratory-transformed line to field cultivars (Figures 1F,G). In this study, we show that inter-chromosomal recombination of *lox* sites can relocate a transgene to a different chromosome, whether to the same location of a homologous chromosome or to another location in a non-homologous chromosome. Although the original target construct must first be introgressed through conventional breeding, subsequent transgenes appended to that locus would be able to use site-specific translocation for linkage drag-free introgression to field cultivars.

## Materials and Methods

### Site-Specific Integration and Rice Transformation

Biolistic-mediated site-specific integration of rice (*Oryza sativa* cv. Zhonghua 11) target line TS131 (Figure 1A) using integrating vector pZH201B has been described (Li et al., 2016) and greater details are available including lines TS325 and TS537 (Figure 1A; Li et al., submitted). Each of these three target lines has a full-length T-DNA construct-expressing reporter gene *gus* (encoding β-glucuronidase), with correct sequence recombination sites, and is located at a non-gene coding DNA >2.9 kb and >0.8 kb from nearest start and stop codons, respectively. Line TS*537* was generated from TS537 through CRISPR/Cas9-mediated mutagenesis (Ma et al., 2015) using oligonucleotides listed in Supplementary Table 1. The primer pair was connected to vector pYLCRISPR/CasPubi-B after annealing. *Agrobacterium*-mediated transformation of rice calluses with a *cre*-expressing construct was conducted as described (Li and Li, 2003). Other genes shown in Figure 1A are *hpt*, *gfp*, and *bar* that encode, respectively, hygromycin phosphotransferase, green fluorescence protein, and bialaphos resistance enzyme.

### Mutated PCR and Restriction Endonuclease Digestion

**FIGURE 2:**
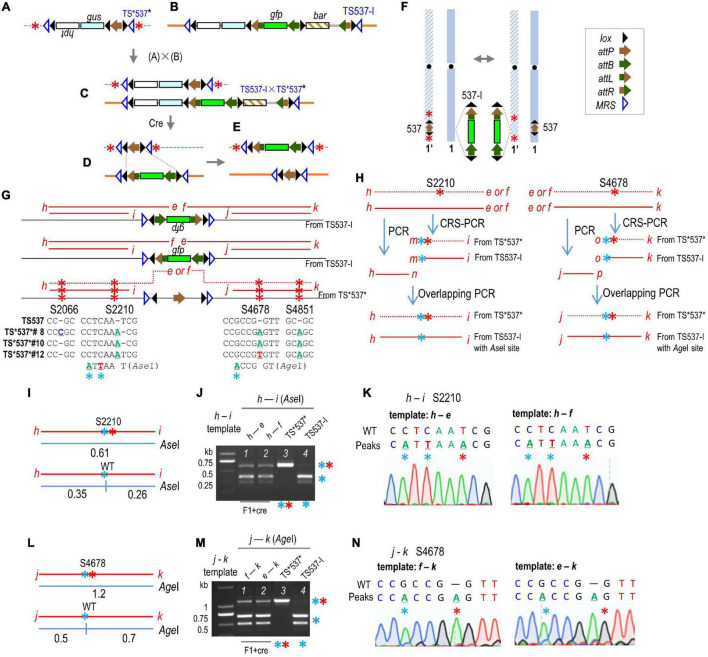
Gene translocation from a target site to an allelic target site. **(A)** TS*537* structure mutated by CRISPR. **(B)** Structure of *gfp-*stacking line TS537-I. **(C)** Structure of F1 hybrid from TS537-I × TS*537*. **(D)** Resolved structure from Cre-mediated deletion; inversion not shown. **(E)** Structure after Cre-mediation reciprocal translocation from recombination between *lox* sites (red dashed lines) from a TS537-I × TS*537* cross **(F)**. **(G)** CRISPR generated mutations in TS*537* (S2066, S2210, S4678, and S4851). Red letters, PCR primers; red lines, PCR products. **(H)** CRS-PCR and overlapping PCR to create *Ase*I or *Age*I restriction site for PCR products derived from TS537, but not from TS*537*. Blue * indicates mutation in PCR primer to form restriction site, red * indicates CRISPR mutations that abolish restriction site. Expected fragment sizes (in kb) and data from *Ase*I **(I,J)** or *Age*I **(L,M)** cleavage of CRS-PCR/overlapping PCR products of F1 + *cre* DNA. Sequencing results of *h–i* fragment resistant to *Ase*I **(K)** or *Age*I **(N)**.

Restriction endonuclease analysis was carried out to distinguish between TS537 and TS*537*. However, because the CRISPR-mediated changes in TS*537* did not destroy an existing restriction site, CRS-PCR (created restriction site PCR) (Qiao et al., 2013; Wang et al., 2016; Avanus and Altınel, 2017; Ding et al., 2017) and overlapping PCR were used to create a restriction site for the TS537 PCR product, but not for the TS*537* PCR product. Specific steps are outlined in Figure 2H as follows: primers *h* + *e*, *h* + *f*, *e* + *k*, and *f* + *k* can only be amplified from F1 + *cre*; one or two bases were changed near the 3′-end of the oligonucleotide *m* or *o*, respectively (Supplementary Figure 1); primers *h* + *n* and *m* + *i* created the overlapping PCR product *h-i*, and similarly, primers *j* + *p* and *o* + *k* created the overlapping PCR product *j–k*. The product *h–i* or *j–k* would be cleaved by *Ase*I or *Age*I (NEB Beijing, China), respectively, if amplified from TS537, but not from TS*537* (Figure 2H).

### PCR and DNA Sequencing

DNA was isolated from ∼100 mg of a 60-day-old rice leaf tissue and ground in liquid nitrogen as described (Lu and Zheng, 1992). PCR was conducted under standard conditions using 1.1 × T3 super PCR mix (Tsingke Biotechnology, Beijing, China) and KOD-FX High-Fidelity DNA Polymerase (TOYOBO, Osaka, Japan) with primers listed in Supplementary Table 2. PCR products were isolated by using HiPure Gel Pure DNA Mini Kit (Magen, Guangzhou, China) and sequenced by Sangon Biotech (Shanghai, China).

## Results and Discussion

### Recombination Between Non-homologous Chromosomes

We sought to test Cre recombinase-mediated recombination of *lox* sites between chromosomes and whether the recombination between sets of flanking inverted *lox* sites could translocate a transgene to another chromosome. Target lines TS131, TS325, and TS537 served as receptor lines, and each harbors a single copy of the target construct (Figure 1A). TS131 is located in the short arm of chromosome 2, TS325 and TS537 are both located in the long arm of chromosome 1, and their chromosome orientation is indicated by the direction of *attP* flanked by the relevant *lox* sites (Figures 1H–K). From Bxb1-mediated integration of the *gfp*-containing plasmid pZH210B (Figure 1B) into TS325 and TS537 (Li et al., submitted), integrant lines TS325-I and TS537-I, respectively, were generated to serve as donor lines (Figure 1C).

The F1 hybrid from an integrant and target line would be expected to harbor two chromosomes with *lox* sites that can recombine with each other if Cre is introduced (Figure 1D). Cre-mediated intra-molecular recombination of *lox* sites is expected to produce a resolved structure (Figure 1E), while inter-chromosomal recombination can generate various intermediates, including one final outcome being the translocation of *gfp* to another chromosome in either orientation (Figures 1F,G). To test this possibility, we conducted four pairs of crosses between homozygous plants, namely, TS325-I♂ × TS131♀(Figure 1H), TS537-I♂ × TS131♀(Figure 1I), TS325-I♂ × TS325♀(Figure 1K). The F1 seeds, hemizygous for two different transgenic loci, were used to induce embryogenic calluses. The *cre* gene was then transformed into these calluses by *Agrobacterium-*mediated gene transfer.

After regeneration of F1 + *cre* plants from calluses, the non-recombined structures were tested by PCR using primers *a* + *f* and *e* + *b* (Figures 1E,L), whereas the translocation of the *gfp*-containing fragment to a different chromosome was also detected by primers *c* + *f* and *e* + *d* (Figures 1F,L). Note that the primers *a*, *b*, *c*, and *d* lie outside the target construct and are, therefore, unique for each chromosome location. Detection by location-specific primers *c* + *f* and *e* + *d*, however, could not distinguish between a double recombination events in the same cell vs. separate recombination events in different cells. Location-specific primers *c* + *d* also failed to amplify a contiguous fragment containing *gfp* that would be ∼4.5, ∼4.9, or ∼4.3 kb from TS131, TS325, or TS537 chromosomes, respectively, likely due to competing reactions of the smaller ∼0.6, ∼1.0, or ∼0.4 kb fragment from the corresponding WT chromosomes. In contrast, the reciprocal product from a translocation, the replacement of the *gfp*-containing fragment by an *attP* fragment, was only 0.8 kb larger than the WT chromosome-derived product. Indeed, location-specific primers *a* + *b* amplified the WT ∼1 kb and a larger ∼1.8 kb band from the TS325-I × TS131- and TS325-I × TS537-derived F1 plants, and a ∼1.2-kb product was detected along with the WT ∼0.4 kb product from the TS537-I × TS131 and TS537-I × TS325 F1 plants (Figure 1M). A contiguous fragment from location-specific primers *a* + *b* indicated a double recombination event from at least some cells.

Since Cre-mediated recombination could also invert *lox*-flanked DNA, the *attP* could be in the opposite orientation in the chromosome (Figure 1G). Using nested PCR of the location-specific *a–b* PCR product, where primer *g* corresponds to within the *attP* sequence (Figures 1E–G), both *attP* orientations were found as amplified products were detected with primers *a* + *g* as well as by primers *g* + *b* (Figure 1M).

### Recombination Between Homologous Chromosomes

To test for potential transgene cassette exchange between homologous chromosomes, it was necessary to have sequence differences in the flanking regions. Therefore, we used CRISPR/Cas9 technology to mutate both sides of TS537 to generate TS*537* (Figure 2A). Out of 96 transgenic plants, 43 had segregated away *cas9*, and 15 of those were sequenced for PCR products from primers *h* + *i* and *j* + *k* (Figure 2G). Three independent TS*537* lines were found with mutations on both sides of the target construct and without heterozygosity, which suggested the same mutations generated in both homolog chromosomes or that the mutations were copied onto its homologous chromosome. Four mutations were found at chromosome 1 positions 35,912,066, 35,912,210, 35,914,678, and 35,914,851, hereafter named sites S2066, S2210, S4678, and S4851, respectively (Figure 2G). TS*537*#8 has mutations at all four sites, whereas TS*537*#10 and TS*537*#12 lack a mutation at S2066. These three lines (i.e., TS*537*#8, TS*537*#10, and TS*537*#12) were crossed with homozygous TS537-I♀ (Figure 2B) to generate F1 heterozygotes (Figure 2C). Cre-*lox* intramolecular recombination is expected to produce a resolved structure (Figure 2D), but inter-chromosomal recombination can also generate various intermediates including the translocation of *gfp* to homologous chromosome (Figures 2E,F). Embryogenic calluses of the F1 heterozygotes were then transformed with a *cre*-expressing construct through *Agrobacterium* infection.

To detect possible chromosome recombination, CRS-PCR and overlapping PCR were used to create a restriction site for the PCR product from TS537-I, but not from TS*537* (Figure 2H and Supplementary Figure 1). Beginning with a template from primers *h* + *e* or *h* + *f*, primers *m* + *i* were then used to change the WT sequence to create an *Ase*I site near S2210 (Figure 2H and Supplementary Figure 1A). Primers *n* and *m* overlap by 23 bp, and the *h–n* and *m–i* fragments were templates for primers *h* + *i*, which would, therefore, have an *Ase*I site if copied from TS537-I, but not from TS*537*. Indeed, the ∼0.61 kb *h–i* fragment (Figure 2I) was cleaved by *Ase*I into 0.35 and 0.26 kb products if from TS537-I DNA, but not from TS*537* DNA (Figure 2J, lanes 3 and 4). Likewise, primers *o* + *k* were used to create an *Age*I site near S4678 (Figure 2L, Supplementary Figure 1B) from template *e–k* or *f–k* (Figure 2H). Primers *p* and *o* overlap by 20 bp, and the *j–p* and *o-k* fragments were templates for primers *j* + *k*. The *j–k* fragment would have an *Age*I site if copied from TS537-I, but not from TS*537*. As shown in Figure 2M, the ∼1.2 kb *j–k* fragments (Figure 2L) were cleaved by *Age*I into 0.5 and 0.7 kb fragments from TS537-I DNA, but not from that of TS*537* DNA (lanes 3 and 4).

From the F1 + *cre* genome, primers *h* + *f* and *e* + *k* should reveal whether *gfp* is linked to WT or mutated DNA (Figure 2G) as primers *e* and *f* were anchored to *gfp*. However, since *gfp* could also be inverted, primers *h* + *e* and *f* + *k* were also tested. These PCR products were then used as templates for nested PCR to amplify *h–i* and *j–k* as described above, followed by *Ase*I or *Age*I treatment to detect mutated sites at S2210 and S4678, respectively (Figure 2H). As shown in the representative data of a 60-day-old F1 + *cre* plant from TS537-I♂ × TS*537*#10♀, some *h–i* products derived from *h–e* and *h–f* templates were immune to *Ase*I cleavage (Figure 2J, lanes 1 and 2). Likewise, some *j–k* products derived from *e–k* and *f–k* templates resisted *Age*I (Figure 2M, lanes 1 and 2). Assuming that the *gfp*-anchored primer sets amplified TS537-I and TS*537-I* equally, and that what translocated across can translocate back, at most 50% of *gfp* DNA would be linked to mutated sites. Based on band intensity, ∼20% of the *gfp* DNA was linked to mutations of the TS*537* genome. The *h–i* and *j–k* fragments that were immune to *Ase*I and *Age*I cleavage (upper bands from Figures 2J,M, lanes 1 and 2) were gel-purified for DNA sequencing. As shown in Figures 2K,N, the predominant peaks show the TS*537*#10 sequence. As these *h–i* and *j–k* fragments were derived from *h–f, h–e, f–k*, and *e–k* templates, with primers *e* and *f* anchored to *gfp*, this demonstrates that the S2210 and S4678 mutations in TS*537*#10 were each linked to *gfp*.

**FIGURE 3:**
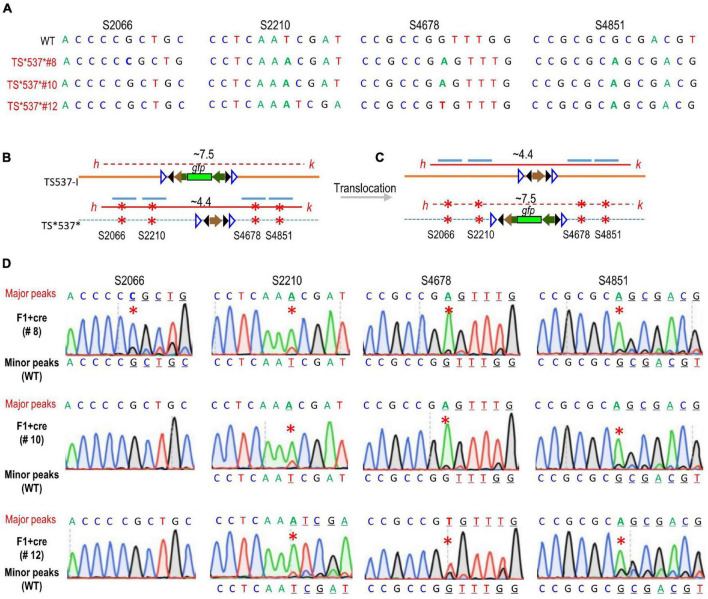
Sequencing results after gene exchange between allelic target sites. **(A)** Sequences of regions corresponding to CRISPR mutation sites. PCR product *h–k* containing *gfp* before **(B)** or after translocation **(C)** was too long (∼7.5 kb) for amplification (dashed red line) in competing reaction with shorter (∼4.4 kb) *attP* fragment (solid red line) without *gfp*. Blue lines depict the four regions sequenced, respectively. **(D)** Sequencing data from the ∼4.4 kb *h–k* fragments from different F1 + *cre* genomes. Major peaks consistent with the TS*537*-derived sequence before translocation **(B)**; minor peaks consistent with the TS537-derived sequence after translocation **(C)**. Red * indicates CRISPR mutations.

Since recombination could generate at 8 genotypes, namely, TS537-I and TS*537* parental types, TS537 and TS*537-I* from cassette exchange, and TS*537-I, TS537*, TS537-I*, and TS*537 from single crossovers, primers *h* + *k* were used to preferentially amplify the smaller ∼4.4 kb size products from TS*537* × TS537-I F1 + *cre* genomic DNA (Figure 3B). Although *lox*-flanked DNA could invert, the regions corresponding to mutated sites should remain constant. In competing reactions, primers *h* + *k* amplified only the ∼4.4 kb *h-k* band with the *attP* site, but not the longer ∼7.5 kb fragment containing *gfp* (Figure 3), and the segments corresponding to the four CRISPR mutations were sequenced. The major peaks were consistent with the TS*537* sequence (CRISPR-mutated sequence) before translocation. Minor peaks were also found that correspond to WT sequence in TS537 or TS537-I (Figure 3C). This would be consistent with translocation of the *attP* site from TS*537* to its homologous chromosome from TS537-I.

To examine whether PCR template switching had been a factor in our analysis, we tested a simulated experiment mixing 1:1 the genomic DNA of TS*537*#8 and TS537-I before PCR with primers *h* + *x* (Supplementary Figure 2). Primer *x* lies within the ampicillin resistance gene in donor vector pZH210B, and the *h–x* band should be ∼3.3 kb, as primer *h* is separated from TS537 by ∼2 kb of genomic DNA. If template switching occurred at a significant rate, S2066 and S2210 mutations should appear as minor peaks. Despite conducting this test under various PCR conditions, minor peaks corresponding to mutations were below detection (Supplementary Figure 2).

### Future Prospects

**FIGURE 4:**
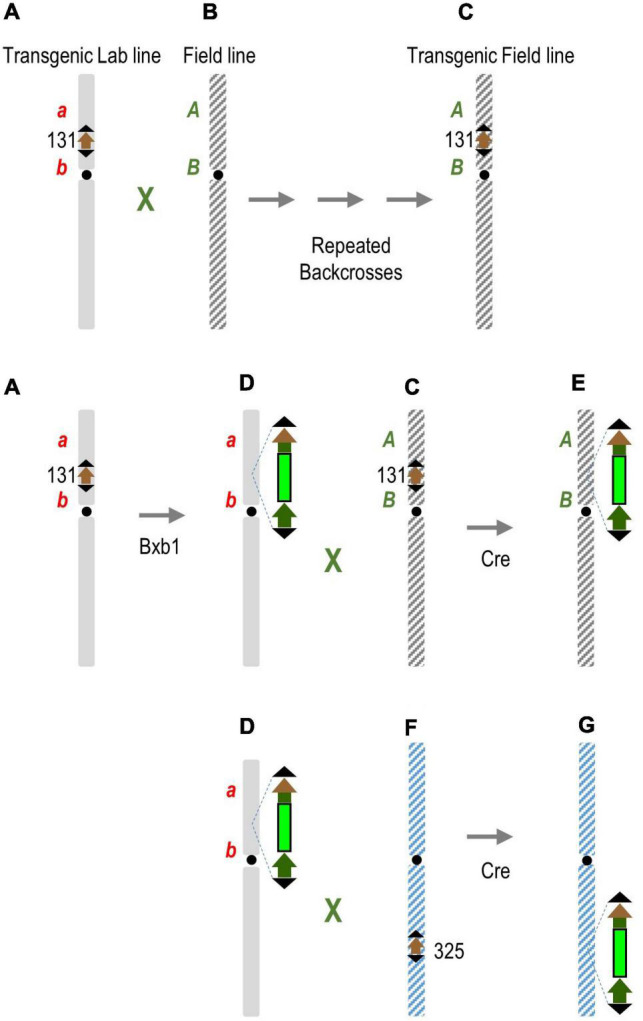
Cre-mediated transgene translocation to break linkage drag. **(A)** Transgenic lab line created by gene transfer, with or without a first transgene (not shown), is crossed repeatedly to a field line **(B)** to introgress the transgenic locus into transgenic field line **(C)** with the combination of elite traits. Subsequent stacking of additional transgenes to the same target site **(D)** can be crossed with the transgenic elite line **(C)**. Introduction of Cre recombinase translocates the new transgenic locus from lab line to field line at the same chromosome position **(E)**. Optionally, Cre translocates the transgenic locus in line **(D)** to a different chromosome **(F)** with a target locus introgressed into a field line **(G)**, which might be necessary if the non-elite traits *a* and/or *b* are too close to the target locus and fail to segregate away. *A*, *B* represent elite traits; *a*, *b* non-elite traits.

In practice, the F1 plant in this exercise would represent a hybrid between a lab cultivar with a newly integrated transgene and a field cultivar previously introgressed with a target site containing already inserted transgenes. To use the Cre-mediated recombination to break genetic linkage on one or both sides of the transgene, the field cultivar must also have a target site. This can be done by conventional breeding (Figures 4A–C) of the target locus with or without a first transgene. Subsequent stacking of additional transgenes would be into the laboratory line (Figure 4D), which can then be crossed with the transgenic field cultivar (Figures 4C,D). Introduction of Cre recombinase would most likely be through a genetic cross, and preferably by a *cre* line already introgressed into elite genotype. Cre would then translocate the new transgenic locus from the lab line to the field line at the same chromosome position (Figure 4E), or optionally, to a different chromosome with a target locus introgressed into a field line (Figures 4F,G). This may be necessary if non-elite traits on either side of the transgene are too close to the target locus to obtain a suitable field target line by conventional breeding. In short, the first introgression is by conventional breeding, while subsequent introgression is facilitated by Cre-*lox-*mediated recombination. This linkage breakage strategy may not be universally applicable, but is suitable for the particular *in planta* gene-stacking method we described (Hou et al., 2014), and this principle can be adapted for other *in planta* gene-stacking schemes.

It is interesting that inter-chromosomal recombination was detected in the F1 generation that had not gone through gamogenesis. This could mean that homologous chromosome synapsis was not a factor, and whether it could further increase the translocation rate remains to be tested. We admit that the sequence data were derived from a population of PCR products; hence, we could not exclude the possibility that some or all fragments had undergone intermolecular recombination on only one side of the donor target site. Nonetheless, breaking linkage drag does not require a cassette exchange reaction if it were between the same homologous chromosome locations, but merely the inter-chromosomal recombination between the transgene and nearby DNA. Beying et al. (2020) reported chromosome arm exchange frequencies at ∼0.01% in *Arabidopsis* somatic cells through the use of CRISPR/Cas9. It is not clear whether the CRISPR/Cas9 reaction is reversible, but Cre definitely catalyzes reversible recombination. Since what translocates across can also translocate back, 100% recombination efficiency would translate to 50% transgene translocation. In this study, transgene translocation reached ∼20%, which ought to be a sufficient rate for recovering progeny with genetic linkages broken. This study did not proceed to the stage of recovering progeny, but since transmission of recombination events and segregation of the *cre* locus have been documented in many previous studies, there is no a prior reason to think these would not be possible.

In summary, this study demonstrates in principle that introducing Cre recombinase into a F1 hybrid serves not only to remove unnecessary DNA such as marker genes and plasmid backbone as previously shown in many studies but could also break genetic linkage on either or both sides of the transgenic locus. Naturally, Cre activity has to be sufficiently efficient in germline cells to insure transmission of recombination events, which means practical implementation of this method would still require the tedious task of testing many different germline-specific promoters for any given crop species.

## Data Availability Statement

The original contributions presented in the study are included in the article/Supplementary Material, further inquiries can be directed to the corresponding authors.

## Author Contributions

DO designed the experiments. QY and RL performed the experiments. QY and DO analyzed the data, discussed results, and wrote the manuscript. All authors approved the final version of the manuscript.

## Conflict of Interest

The authors declare that the research was conducted in the absence of any commercial or financial relationships that could be construed as a potential conflict of interest.

## Publisher’s Note

All claims expressed in this article are solely those of the authors and do not necessarily represent those of their affiliated organizations, or those of the publisher, the editors and the reviewers. Any product that may be evaluated in this article, or claim that may be made by its manufacturer, is not guaranteed or endorsed by the publisher.
