# Supplementary material for: Site-Specific Sequence Exchange Between Homologous and Non-homologous Chromosomes
Source: Front Plant Sci. 2022 Feb 3;13:828960. doi: 10.3389/fpls.2022.828960 (PMC8850970; doi:10.3389/fpls.2022.828960)
Supplement: Supplementary file 1 [file Table_1.DOCX]

**Supplementary Table 1.** Primers for CRISPR

| Site | primer | sequence |  |
| --- | --- | --- | --- |
| S2066 | F | tcaGAGCGTGACAAGCAGCGGGG | U6c target |
|  | R | aaacCCCCGCTGCTTGTCACGCT | U6c target |
| S2210 | F | ggcaCCAAACTCTCGGATCGATTG | U3 target |
|  | R | aaacCAATCGATCCGAGAGTTTGG | U3 target |
| S4678 | F | gccGCACGTCTACACCAAACCGG | U6a target |
|  | R | aaacCCGGTTTGGTGTAGACGTG | U6a target |
| S4851 | F | gttGCACGTCAAAACGTCGCGCG | U6b target |
|  | R | aaacCGCGCGACGTTTTGACGTG | U6b target |

**Supplementary Table 2.** Primers for PCR

| primer | sequence |  |
| --- | --- | --- |
| *e* | gggcgaattttgcgacaacatgtcg |  |
| *f* | cagtgttgctagccaggatttaaga |  |
| *g* | gttatcagtggtttgtctgg |  |
| *h* | tacctcgcgaactgaccgtc |  |
| *i* | cagcatacatctatacattc |  |
| *j* | atgtgtgtatgacaggtaggatc |  |
| *k* | ttagtactacatcgatccatctc |  |
| *m* | gcttataaatcttccgagtaggcaagcattaa |  |
| *n* | gcctactcggaagatttataagc |  |
| *o* | gcggtgatcgattggtggccaccg |  |
| *p* | ggccaccaatcgatcaccgc |  |
| *x* | tatggatgaacgaaatagac |  |
| *131-f* | tacacataatattaagcacacccgg | Outside TS131, could be *c* |
| *131-r* | catttgttaatgctttcactgacc | Outside TS131, could be *d* |
| *325-f* | cgtaccatgcaagacacatcca | Outside TS325, could be *a* or *c* |
| *325-r* | cacattagctctgctctaggttca | Outside TS325, could be *b* or *d* |
| *537-f* | ccgtactatagtaattctgtagc | Outside TS537, could be *a* or *c* |
| *537-r* | cctggacgaggagttgtgctt | Outside TS537, could be *b* or *d* |
