# Supplementary material for: Site-Specific Sequence Exchange Between Homologous and Non-homologous Chromosomes
Source: Front Plant Sci. 2022 Feb 3;13:828960. doi: 10.3389/fpls.2022.828960 (PMC8850970; doi:10.3389/fpls.2022.828960)
Supplement: Supplementary file 2 [file Data_Sheet_1.PDF]

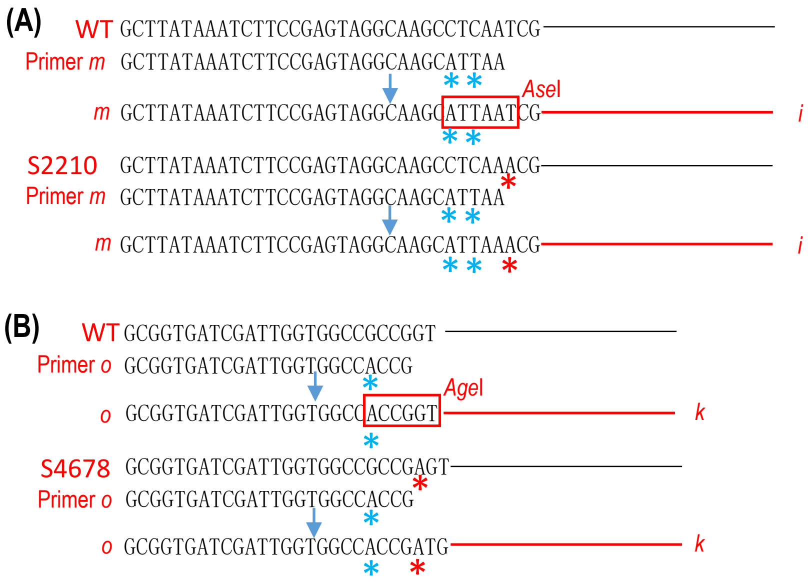


**Supplementary Figure 1.** Primers for CRS (created restriction site) PCR (**A**) Primer *m* forms *Ase*I site in PCR from wild-type template but not from template with CRISPR mutation S2210. (**B**) Primer *o* forms *Ase*I site in PCR from wild-type template but not from template with CRISPR mutation S4678.

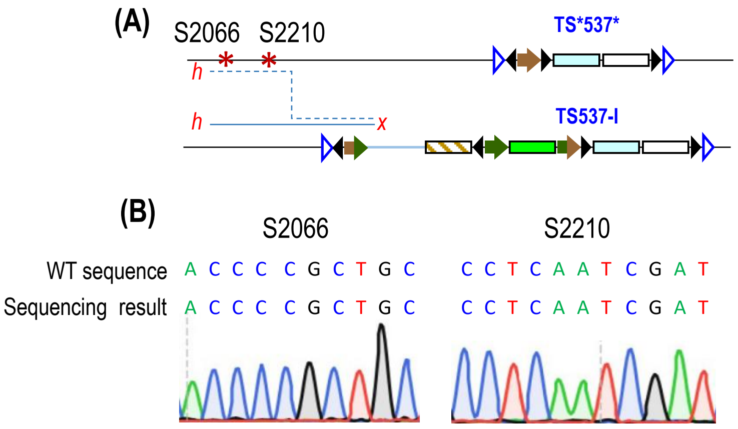


**Supplementary Figure 2.** Primers *h*+*x* tested for template switching. (**A**) PCR with primer *h*+*x* using the 1:1 mixture of TS*537* and TS537-I genomic DNA as template. The *h*-*x* full line fragment represents the normal PCR product that can be amplified. Primer *x* primes only from TS537-I genome, and *h*-*x* dotted line indicates expected product if template switching had occurred. (**B**) Sequencing data of *h*-*x* PCR product shows wild-type sequence, without minor peaks to show the presence of S2066 and S2210 mutations.
